# Effects of patient age on patency of chronic hemodialysis vascular access

**DOI:** 10.1186/s12882-019-1604-7

**Published:** 2019-11-21

**Authors:** Seonjeong Jeong, Hyunwook Kwon, Jai Won Chang, Min-Ju Kim, Khaliun Ganbold, Youngjin Han, Tae-Won Kwon, Yong-Pil Cho

**Affiliations:** 1 Division of Vascular Surgery, Department of Surgery, University of Ulsan College of Medicine and Asan Medical Center, 88, Olympic-ro 43-gil, Songpa-gu, Seoul, 05505 Republic of Korea; 2 Division of Nephrology, Department of Internal Medicine, University of Ulsan College of Medicine, Asan Medical Center, Seoul, Republic of Korea; 3 Department of Clinical Epidemiology and Biostatistics, University of Ulsan College of Medicine, Asan Medical Center, Seoul, Republic of Korea; 4 Department of Surgery, Mongolian National University of Medical Sciences, Ulaanbaatar, Mongolia

**Keywords:** Age, arteriovenous fistula, arteriovenous graft, Chronic kidney disease, renal dialysis

## Abstract

**Background:**

In this single-center, retrospective observational study, we assessed the long-term patency of vascular access (VA) after first VA placement to uncover independent risk factors associated with VA patency in Asian hemodialysis (HD) patients stratified by age. We also investigated factors associated with VA patency among older HD patients according to the type of VA in the overall study population.

**Methods:**

The study period was from January 2011 to December 2013. A total of 651 chronic HD patients with confirmed first upper-extremity VA placement were enrolled, and their records were analyzed retrospectively. A total of 445 patients (68.4%) made up the nonelderly group (< 65 years), and 206 patients (31.6%) were in the elderly group (≥ 65 years). Study outcomes were defined as primary or secondary VA patency.

**Results:**

Autologous arteriovenous fistula (AVF) was more common in the nonelderly group (*P* <  0.01). Kaplan–Meier curve survival analysis indicated that primary patency was longer in the nonelderly group (*P* <  0.01); secondary patency, however, was similar between groups (*P* = 0.37). The multivariate analysis of factors associated with primary VA patency revealed that increased age (hazard ratio [HR], 1.02; 95% confidence interval [CI], 1.01–1.03; *P* <  0.01) was associated with shorter primary patency, and AVF (HR, 0.38; 95% CI, 0.28–0.51; *P* <  0.01) was associated with longer primary patency. AVF (HR, 0.57; 95% CI, 0.37–0.87; *P* = 0.010) and diabetes mellitus (HR, 1.56; 95% CI, 1.07–2.29; *P* = 0.02) were independently associated with longer and shorter secondary patency periods, respectively; however, increased age was not a risk factor for decreased secondary patency.

**Conclusions:**

Increased age was associated with shorter primary patency but not secondary patency, whereas AVF placement was associated with longer primary and secondary patency. Considering the similar rates of secondary patency between groups and the superior patency of AVF compared to arteriovenous graft, a fistula-first strategy should be applied to appropriate older patients.

## Background

The number of patients over 65 years of age diagnosed with chronic kidney disease (CKD) and requiring renal replacement therapy has been steadily increasing [1]. Although fistula-first is the recommended strategy for all hemodialysis (HD) patients [2–4], and age has not been found to be a significant contributing factor to the patency of functioning autologous arteriovenous fistulas (AVFs) across several studies [5–8], controversy remains regarding whether fistula-first is appropriate for elderly HD patients [9–13]. AVF placement in elderly patients is more challenging because of their relatively higher incidence of comorbidities and operative risks, longer AVF maturation times, limited life expectancies, and recent data indicating a lack of a survival benefit compared with arteriovenous graft (AVG) or central venous catheter (CVC) use [8–10, 14–16]. Factors influencing management decisions in older HD patients, including the optimal type of vascular access (VA), differ from considerations for younger patients.

This study compared long-term VA patency in an Asian HD patient population with confirmed first VA placement stratified by age (< 65 years vs. ≥ 65 years) and evaluated potential independent risk factors associated with VA patency in these patients. We also investigated factors associated with VA patency among subgroups of patients 65 years and older and according to VA type in the overall study population.

## Methods

### Study design and patient population

This single-center, observational study was conducted retrospectively using data extracted from the medical records of chronic HD patients. The study protocol was approved by the Institutional Review Board (2018–1318) at our hospital, which waived the need for informed consent because of the retrospective nature of the study.

A total of 876 patients aged 20 years and older with confirmed first upper-extremity VA placement for HD at our hospital between January 1, 2011, and December 31, 2013 were screened for this study. Of these, 694 with AVFs (79.2%) and 182 with AVGs (20.8%) were collected. We excluded patients lost to follow up (*n* = 89, 10.2%) and those with a malignancy (*n* = 136, 15.5%). A final total of 651 HD patients (74.3%) was stratified by patient age with the nonelderly group including patients less than 65 years and the elderly group including patients at least 65 years at the time of VA placement. Data were analyzed retrospectively. We then examined the association between clinical variables and outcomes in the elderly group using Cox proportional hazard regression models. We also evaluated the association between clinical variables and outcomes according to AVF versus AVG VA. The elderly group was then subdivided for subgroup analyses into those from 65 to 74 years of age and those at least 75 years of age. In our study population, a nephrologist was involved with each patient for all medication adjustments, planning of VA type and HD initiation, and VA surveillance [17].

### Index procedures and definitions

The preferred option for VA placement is AVF, followed by AVG, at the most distal site of the nondominant arm that satisfies necessary criteria for vessel suitability, as evaluated by physical examination alone or with supplemental duplex ultrasound. All VA placement procedures were performed under local anesthesia by specially trained vascular surgeons, as described in previous publications [17–20]. VA types were categorized as AVF (forearm or upper arm) or AVG (straight or U-shaped forearm graft or straight upper arm graft). Postoperative surveillance was conducted in accordance with the clinical practice guidelines of the Society for Vascular Surgery regarding surgical placement and maintenance of arteriovenous HD access [17, 21].

VA performance was defined as described in previous publications [17–21]. A functioning VA allowed for at least six adequate HD sessions with successful two-needle cannulation without VA-related complications. Primary VA patency was defined from the time of VA placement until the first intervention to preserve or restore blood flow, first VA failure, or study end, whichever occurred first. Secondary VA patency was defined from the time of VA placement until VA abandonment for any cause, regardless of the number of subsequent interventions [17, 18, 22, 23]. Early mortality was defined as all-cause mortality that occurred within 3 months of VA placement but prior to its use, with HD maintained via CVC. AVF maturation failure was defined as an AVF inadequate for successful needle cannulation after placement [23, 24]. Early thrombosis was defined as the absence of thrill or flow on duplex ultrasound or fistulogram within 30 days of HD initiation via a functioning VA [25]. Body mass index (BMI) was defined as weight in kg divided by height in m^2^ at the time of VA placement. Peripheral arterial occlusive disease (PAOD) was defined as a previous history of any therapeutic interventions for PAOD or an ankle-brachial index less than or equal to 0.9 as measured by Doppler ultrasound [26].

### Study outcomes and follow-up

Study outcomes of interest were primary and secondary VA patency. Formal follow-up visits by clinical examination alone or with supplemental duplex ultrasound were conducted at the Vascular Surgery out-patient clinic to assess VA performance at 1 and 6 weeks after VA placement. Once stability was established, clinical surveillance at our facility was terminated. Follow-up visits with laboratory assessments at the Nephrology out-patient clinic were planned at approximately 6-month intervals, and the latest follow-up data were obtained from medical records or follow-up physicians. For patients receiving follow-ups at other centers, telephone interviews with the patients or their family members were conducted to obtain information about each patient’s general health status, the function of the original VA, and all diagnostic and therapeutic interventions during the interim. Risk factors of interest, clinical characteristics, and long-term clinical outcomes for all patients were recorded in an Excel database (Microsoft Corp., Redmond, WA, USA) and analyzed retrospectively.

### Statistical analysis

Data were recorded in an Excel database, and patients in the nonelderly and elderly groups were compared. Categorical variables are reported as frequencies or percentages, and continuous variables as means or standard deviations. Differences between the two groups were assessed using the chi-squared test for categorical variables and Student’s *t*-test for continuous variables. Long-term event-free rates were analyzed by Kaplan–Meier curve. Long-term event-free rates were compared between patients less than 65 and at least 65 years, with estimations calculated using the log-rank test. Univariate and multivariate analyses of the associations between clinical variables and study outcomes (primary and secondary VA patency) were performed with Cox proportional hazards modeling. This analysis utilized the event of interest and the time interval from VA placement to the date of the event or last follow-up as the outcome. We adapted univariate Cox proportional hazard regression models to calculate hazard ratios (HRs) with 95% confidence intervals (CIs). Using the Cox regression model, we evaluated the associations between clinical variables and outcomes. Variables with a *P*-value of less than 0.1 on univariate analysis were included in the multivariate analysis. A *P*-value of less than 0.05 was considered statistically significant. Statistical analyses were performed with SPSS version 21.0 (IBM Corp., Armonk, NY, USA).

## Results

The study cohort consisted of 651 chronic HD patients with first VA placements from our hospital, stratified by age into nonelderly (*n* = 445, 68.4%) and elderly (*n* = 206, 31.6%) groups. No mortality or morbidity was associated with VA placement. The baseline and clinical characteristics of the study population are presented in Table 1. AVF placement was performed more often in the nonelderly group (*P* <  0.01) than the elderly group. The elderly group had a higher prevalence of atherosclerotic risk factors and comorbidities than the nonelderly group. The proportion of patients taking antiplatelet medications (*P* <  0.01) and of those with early mortality (*P* <  0.01) were significantly higher in the elderly group, whereas there were no significant differences in AVF maturation failure (*P* = 0.80) or early VA thrombosis (*P* = 0.16) between the two groups.

**Table 1:** Baseline demographics and clinical characteristics of the study population at the time of VA placement according to patient age

	< 65 years *n* = 445	≥65 years *n* = 206	*P*-value
Age (years)	50.0 ± 10.2	72.7 ± 5.5	< 0.01
Female sex	169 (38.0)	92 (44.7)	0.11
BMI (kg/m^2^)	23.3 ± 3.6	23.1 ± 3.7	0.46
Type of VA			
AVF	398 (89.4)	123 (59.7)	< 0.01
Forearm	206 (51.8)	58 (47.2)	0.37
Upper arm	192 (48.2)	65 (52.8)	
AVG	47 (10.6)	83 (40.3)	
Forearm	24 (51.1)	25 (30.1)	0.02
Upper arm	23 (48.9)	58 (69.9)	
On hemodialysis	232 (52.1)	127 (61.7)	0.02
Underlying diseases			
Hypertension	373 (83.8)	181 (87.9)	0.18
DM	200 (44.9)	124 (60.2)	< 0.01
Smoker	105 (23.6)	44 (21.4)	0.53
CVD	55 (12.4)	71 (34.5)	< 0.01
CVA	31 (7.0)	43 (20.9)	< 0.01
PAOD	19 (4.3)	17 (8.3)	0.04
Medications			
Anti-platelets	184 (41.3)	122 (59.2)	< 0.01
Anti-coagulants	28 (6.3)	11 (5.3)	0.63
Early mortality^a^	12 (2.7)	16 (7.8)	< 0.01
Maturation failure^b^	26 (5.8)	11 (5.3)	0.80
Early thrombosis^c^	12 (2.7)	10 (4.9)	0.16

Continuous data are expressed as mean ± standard deviation, and categorical data as number (%)

*AVF* Autologous arteriovenous fistula, *AVG* Arteriovenous graft, *BMI* Body mass index, *CVA* History of cerebrovascular accident, *CVD* Cardiovascular disease, *DM* Diabetes mellitus, *PAOD* Peripheral arterial occlusive disease, *VA* Vascular access

^a^ All-cause mortality within 3 months of VA placement but before use

^b^ AVF maturation failure

^c^ Absence of thrill and/or flow within 30 days of hemodialysis initiation via a functioning VA

**Fig. 1:**
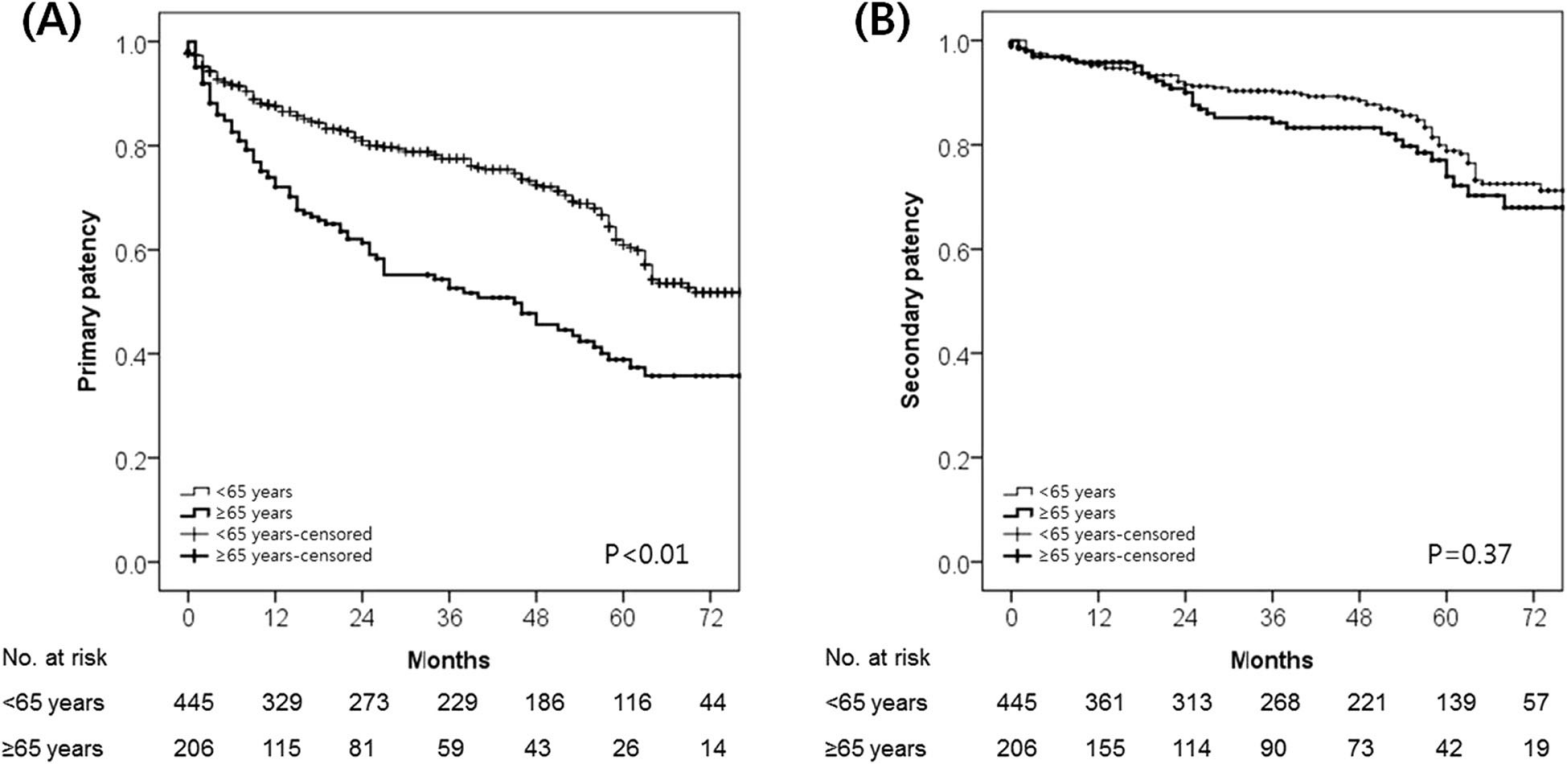
Kaplan–Meier survival analysis. Kaplan–Meier estimates of (**a**) primary and (**b**) secondary patency rates in the study population stratified by age (< 65 years vs. ≥65 years)

A Kaplan–Meier survival analysis revealed that primary VA patency was significantly longer in the nonelderly group (*P* <  0.01), whereas secondary patency was similar between the two groups (*P* = 0.37) (Fig. 1). Elderly patients had a reduced primary patency rate at all time points compared with nonelderly patients. The mean durations of primary and secondary VA patency for the nonelderly group were 62.9 months (95% CI, 59.6–66.2 months) and 75.1 months (95% CI, 72.4–77.8 months), respectively. For the elderly group, these values were 47.6 months (95% CI, 41.6–53.5 months) and 74.9 months (95% CI, 69.9–79.9 months), respectively.

Clinical variables associated with study outcomes were analyzed using univariate and multivariate Cox proportional hazards regression. In the adjusted models, increased age (HR, 1.02; 95% CI, 1.01–1.03; *P* <  0.01) was significantly associated with shorter primary patency, whereas type of VA (AVF) (HR, 0.38; 95% CI, 0.28–0.51; *P* <  0.01) was a positive predictor for longer primary patency (Table 2). An analysis of the associations between clinical variables and secondary VA patency was conducted, and AVF (HR, 0.57; 95% CI, 0.37–0.87; *P* = 0.010) and diabetes mellitus (DM) (HR, 1.56; 95% CI, 1.07–2.29; *P* = 0.02) were independently associated with longer or shorter secondary patency, respectively. Of note, increased age was not associated with secondary patency duration (Table 3). In our analysis, female sex was not a significant risk factor in primary or secondary VA patency, probably due to the higher proportion of AVG placements in females than males; there were 190 (26.5%) AVF female patients and 331 (63.5%) AVF male patients.

**Table 2:** Factors associated with primary VA patency

	Univariate		Multivariate	
	HR (95% CI)	*P*-value	HR (95% CI)	*P*-value
Increased age	1.03 (1.02–1.04)	< 0.01	1.02 (1.01–1.03)	< 0.01
Female sex	1.04 (0.80–1.35)	0.76	NA	NA
BMI	0.97 (0.94–1.01)	0.15	NA	NA
AVF	0.32 (0.24–0.42)	< 0.01	0.38 (0.28–0.51)	< 0.01
Hypertension	0.99 (0.70–1.40)	0.94	NA	NA
DM	1.41 (1.09–1.82)	< 0.01	1.17 (0.90–1.53)	0.25
Smoker	0.99 (0.73–1.34)	0.93	NA	NA
CVD	1.30 (0.95–1.78)	0.10	NA	NA
CVA	1.17 (0.79–1.72)	0.44	NA	NA
PAOD	1.00 (0.58–1.00)	0.99	NA	NA

*AVF* Autologous arteriovenous fistula, *BMI* Body mass index, *CI* Confidence interval, *CVA* History of cerebrovascular accident, *CVD* Cardiovascular disease, *DM* Diabetes mellitus, *HR* Hazard ratio, *NA* Not applicable, *PAOD* Peripheral arterial occlusive disease, *VA* Vascular access

**Table 3:** Factors associated with secondary VA patency

	Univariate		Multivariate	
	HR (95% CI)	*P*-value	HR (95% CI)	*P*-value
Increased age	1.01 (1.00–1.03)	0.104	NA	NA
Female sex	0.93 (0.63–1.38)	0.723	NA	NA
BMI	1.00 (0.95–1.06)	0.884	NA	NA
AVF	0.53 (0.35–0.82)	0.004	0.57 (0.37–0.87)	0.010
Hypertension	0.96 (0.58–1.59)	0.877	NA	NA
DM	1.64 (1.12–2.40)	0.011	1.56 (1.07–2.29)	0.02
Smoker	1.13 (0.74–1.73)	0.581	NA	NA
CVD	1.29 (0.82–2.05)	0.272	NA	NA
CVA	1.32 (0.76–2.27)	0.323	NA	NA
PAOD	0.81 (0.33–1.98)	0.643	NA	NA

*AVF* Autologous arteriovenous fistula, *BMI* Body mass index, *CI* Confidence interval, *CVA* History of cerebrovascular accident, *CVD* Cardiovascular disease, *DM* Diabetes mellitus, *HR* Hazard ratio, *NA* Not applicable, *PAOD* Peripheral arterial occlusive disease, *VA* Vascular access

**Fig. 2:**
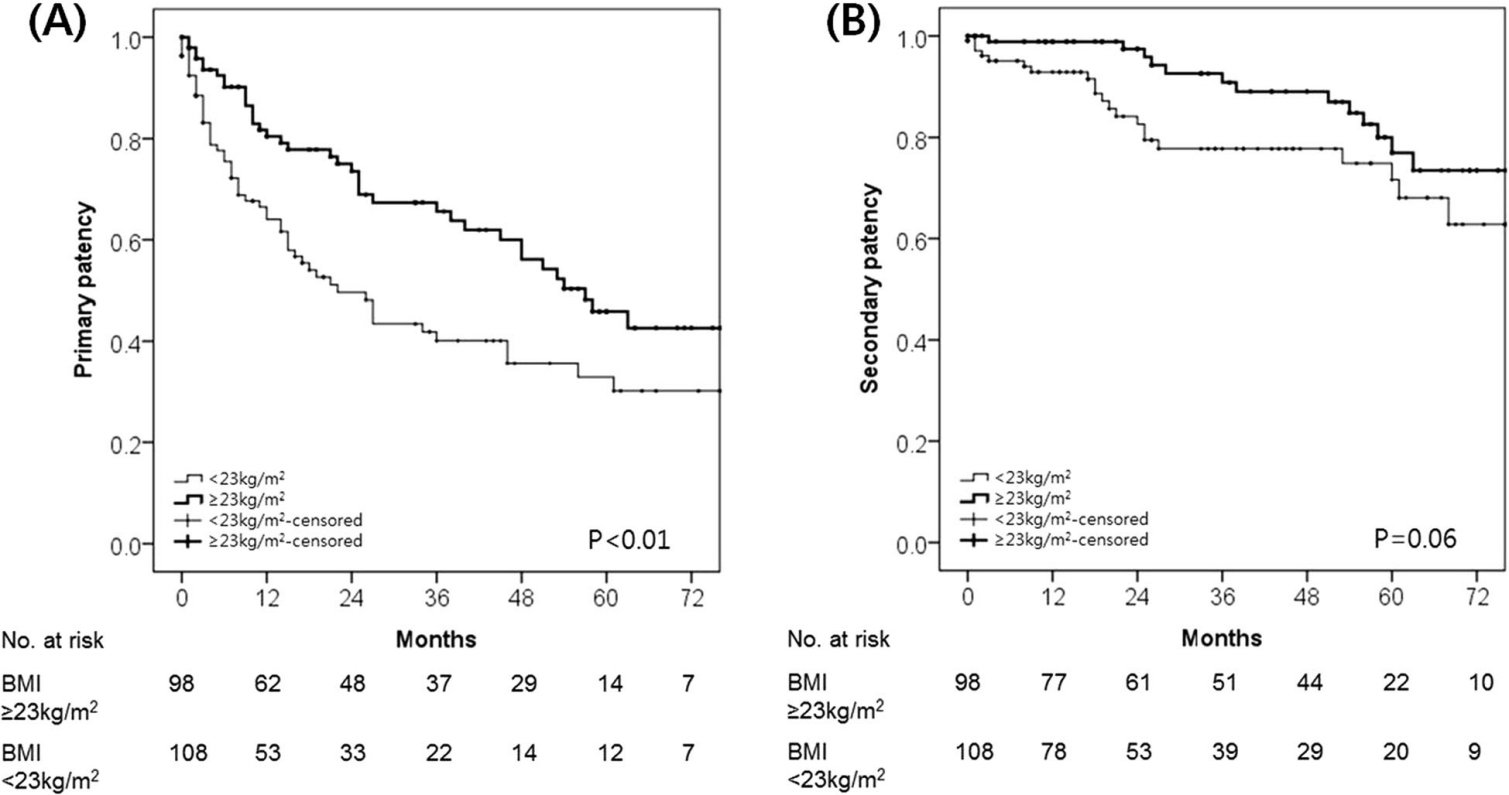
Kaplan–Meier survival analysis of elderly patients (≥65 years). Kaplan–Meier estimates of (**a**) primary and (**b**) secondary VA patency rates in elderly patients stratified by body mass index (BMI) (< 23 kg/m^2^ vs. ≥23 kg/m^2^)

We performed subgroup analyses of the associations between clinical variables and study outcomes in the elderly group (*n* = 206) (Additional file 1: Tables S1 and S2). Multivariate analysis of factors associated with VA patency indicated that a higher BMI was independently associated with longer primary patency (HR, 0.93; 95% CI, 0.88–0.99; *P* = 0.02). Results also demonstrated that AVF (HR, 0.36; 95% CI, 0.24–0.54; *P* <  0.01) was independently associated with longer primary patency. Increased age was associated with shorter secondary patency (HR, 1.13; 95% CI, 1.07–1.20; *P* <  0.01). A Kaplan–Meier survival analysis of elderly patients stratified by BMI (< 23 kg/m^2^ vs. ≥ 23 kg/m^2^) revealed that higher BMI was significantly correlated with longer primary VA patency (*P* <  0.01) and trended towards longer secondary patency (*P* = 0.06) (Fig. 2). Patients with a BMI of at least 23 kg/m^2^ presented with an increased primary patency rate at all time points compared to those with a BMI below 23 kg/m^2^.

We also performed subgroup analyses of the associations between clinical variables and outcomes according to the type of VA (AVF or AVG). Among patients with AVF placement (*n* = 521), increased age was the only risk factor associated with shorter primary AVF patency (HR, 1.01; 95% CI, 1.00–1.02; *P* = 0.08) (Additional file 1: Table S3). DM with AVF showed a trend towards shorter secondary patency (HR, 1.48; 95% CI, 0.96–2.28; *P* = 0.08) (Additional file 1: Table S4). Among patients with AVG placement (*n* = 130), increased age was the only risk factor significantly associated with shorter primary (HR, 1.02; 95% CI, 1.00–1.04; *P* = 0.04) or secondary (HR, 1.04; 95% CI, 1.00–1.07; *P* = 0.03) AVG patency, whereas increased BMI was significantly associated with a longer primary AVG patency (HR, 0.92; 95% CI, 0.87–0.98; *P* = 0.01) and showed a similar trend toward longer secondary patency (HR, 0.91; 95% CI, 0.82–1.01; *P* = 0.08) (Additional file 1: Tables S5 and S6).

We performed another subgroup analysis based on age (65–75 years vs. ≥75 years) in the elderly group (≥65 years, *n* = 206). We found that AVF placement was more often performed in patients aged 65–75 years (*P* <  0.01). Patients aged 75 years and older had a higher prevalence of atherosclerotic risk factors and comorbidities than the 65–75 group. No significant differences in the proportion of patients taking antiplatelet medications (*P* = 0.77), or with early mortality (*P* = 0.26), or maturation failure (*P* = 0.19) were observed between the two elderly patient subgroups, whereas early VA thrombosis was significantly higher in patients older than 75 years (*P* = 0.03) (Additional file 1: Table S7). The mean durations of primary and secondary VA patency for the patients aged 65–75 years were 52.6 months (95% CI, 45.3–59.9 months) and 79.8 months (95% CI, 74.4–85.2 months), respectively. For those ≥75 years, mean durations were 36.9 months (95% CI, 41.6–53.5 months, *P* = 0.013) and 62.4 months (95% CI, 53.9–70.9 months, *P* <  0.01), respectively. In the adjusted models of patients aged 75 years and older, type of VA (AVF) was the only positive predictor of longer primary patency (HR, 0.31, 95% CI, 0.14–0.67; *P* <  0.01) (Additional file 1: Table S8), whereas increased age was the only risk factor significantly associated with shorter secondary patency (HR, 1.20; 95% CI, 1.06–1.36; *P* = 0.01). The type of VA (AVF vs. AVG) was not associated with secondary patency duration (HR, 1.93, 95% CI, 0.60–6.20; *P* = 0.27) (Additional file 1: Table S9).

## Discussion

Many patient-level and intervention-specific factors have been suggested as potential predictors of shorter VA patency duration. Although some studies have suggested that VA patency is shorter in women, the elderly, and individuals with DM [27], others have argued that increased age should not be a limiting factor when considering VA options for HD, as similar survival and AVF patency rates were observed for younger and older patients [5–8, 28]. Inconclusive findings across multiple studies highlight the fact that the maintenance of functional VA is a complex and dynamic process, requiring close collaboration among the vascular surgeon, nephrologist and nursing staff to ensure successful VA placement and two-needle cannulation, an adequate HD session, compression for hemostasis, and surveillance. The presence and severity of patient-level factors can also influence the process.

Ethnic differences in environmental and genetic factors, comorbidities, and other characteristics might also influence VA utilization patterns [29]. Although this study cohort consisted of only Korean Asians and is not representative of other ethnic groups, this limitation is also a unique feature of this study, because there is little available data on the association of ethnicity with VA outcomes [30]. The major finding of this study involving an Asian population with CKD requiring HD was that primary VA patency was significantly longer in the nonelderly group, whereas secondary patency was similar between the two groups despite a higher prevalence of atherosclerotic risk factors and comorbidities in the elderly group. Increased age was significantly associated with shorter primary patency but did not significantly affect secondary patency. In the subgroup analyses of the elderly patient group, AVF placement was associated with longer primary VA patency; subgroup analyses according to the type of VA indicated increased age as a significant risk factor for shorter primary AVF patency in this sub-cohort and shorter primary and secondary AVG patency.

Considering advanced age-related complications, patient age is an important factor to consider when determining the ideal type of VA [30]. Reduced life expectancy with increasing age is another competing risk factor for AVF maturation time and use [31]. These issues make it challenging to balance among the factors of the likelihood of patient survival, functional VA survival, and relevant potential complications [30]. The complexities involved in planning VA placement were highlighted in a study of octogenarians who underwent vein mapping preoperatively during the predialysis period and received an AVF [16]; in that cohort, 32% died before starting HD, and 57.5% of patients died within 18 months of starting HD. Of the patients who died, 55.5% died within 6 months of starting HD; 70% of them had AVFs placed, and there were no matured AVFs available for cannulation prior to the patients’ deaths [16]. Although old age alone should not preclude AVF placement because AVFs are likely appropriate for relatively healthy elderly patients with few comorbidities, AVGs may be more suitable for elderly patients with limited life expectancies [9, 16, 30, 32–34]. Age and comorbidities were associated with additive and higher risks for shorter functional VA patency. Our analysis indicated that although primary VA patency was significantly longer among nonelderly patients, there were no differences in AVF maturation failure and secondary VA patency between the nonelderly and elderly patients. In our study population, all patients had a nephrologist involved in the planning of VA placement and patient care, and we maintained a strategy of aggressive endovascular and surgical intervention to preserve and restore the patency of failing or failed VAs [17, 34, 35]. We speculate that the planning of VA placement in collaboration with a nephrologist and our aggressive management strategy for dysfunctional VA reduced AVF maturation failure and resulted in the similar secondary VA patency durations observed in the nonelderly and elderly patients. Recent studies and other commentaries may offer additional guidance regarding the planning of VA placement according to factors and conditions associated with old age, including palliative care [30, 36–39].

In the general population, the deleterious effects of obesity on patient survival are well known [40, 41]; however, several studies have consistently described a survival benefit of a high BMI for chronic HD patients [42–46]. In a study of 1486 patients, 340 of whom were obese, only the morbidly obese patients (BMI ≥35 kg/m^2^) had an increased risk of AVF maturation failure [47]; obesity in and of itself was not associated with AVF patency. In our analysis, higher BMI within the normal range was related to longer primary VA patency among elderly patients, and among those with AVGs, higher BMI was significantly associated with longer primary AVG patency and trended toward longer secondary patency. No association between BMI and VA patency was observed in patients with AVFs. Although the recent Westernization of dietary habits has led to increasing BMIs in Asian countries, Asian populations are overall less obese than their Western counterparts. We speculated that the patients who received AVGs had poorer general health status than the patients who received AVFs, and our findings might reflect the generally healthier status of chronic HD patients with higher BMIs compared to the health status of those with lower BMIs.

Our study had some limitations, in particular concerning the retrospective design and small sample size from a single-center cohort. We acknowledge potential selection and information biases on the part of the physicians or patients; indication bias and patient self-selection may also have influenced our findings. Decisions about the type of VA were mainly made by the physician based on the expected level of vessel diameter and quality and the estimated risk of AVF maturation failure. We also acknowledge other important factors could have impacted the results that were not available in our data sources, such as uremic signs and symptoms at the time of HD initiation, exact time of CVC exposure, and vessel diameter and quality; these might have accounted for the differences in outcomes we observed compared to other studies. Finally, our study cohort consisted of only Korean Asians, and our findings may not be generalizable to other Asians or other ethnic groups.

## Conclusions

Considering the similar secondary VA patency periods in the nonelderly and elderly groups, and the superior patency of AVFs compared with AVGs, a fistula-first strategy is appropriate for elderly patients who are good candidates for AVF placement based on patient demographics (atherosclerosis risk factors and comorbidities) and vessel diameter and quality as evaluated by physical examination alone or with supplemental ultrasound. In our study population, all patients had a nephrologist involved in planning and patient care from the predialysis stage onward. We expect that collaboration with other specially trained medical staffs and their engagement in the consensus process will further improve VA patency in elderly patients.

## Supplementary information

**Additional file 1: Table S1.** Factors associated with primary VA patency in subgroup analyses of patients aged 65 years and older. This table contains the associations between clinical variables and primary VA patency in the elderly group. **Table S2.** Factors associated with secondary VA patency in subgroup analyses of patients aged 65 years and older. This table contains the associations between clinical variables and secondary VA patency in the elderly group. **Table S3.** Factors associated with primary patency in subgroup analyses of patients with AVF placement. This table contains the associations between clinical variables and primary patency in patients with AVF placement. **Table S4.** Factors associated with secondary patency in subgroup analyses of patients with AVF placement. This table contains the associations between clinical variables and secondary patency in patients with AVF placement. **Table S5.** Factors associated with primary patency in subgroup analyses of patients with AVG placement. This table contains the associations between clinical variables and primary patency in patients with AVG placement. **Table S6.** Factors associated with secondary patency in subgroup analyses of patients with AVG placement. This table contains the associations between clinical variables and secondary patency in patients with AVG placement. **Table S7.** Baseline demographics and clinical characteristics of the ≥65 study population at the time of VA placement. This table contains baseline demographic and clinical characteristics of subgroup analyses based on age (65–75 years vs. ≥75 years) in the elderly group. **Table S8.** Factors associated with primary VA patency in subgroup analyses of patients ≥75. This table contains the associations between clinical variables and primary patency in patients ≥75. **Table S9.** Factors associated with secondary VA patency in subgroup analyses of patients ≥75. This table contains the associations between clinical variables and secondary patency in patients ≥75.

## Data Availability

The datasets used and/or analyzed during the current study are available from the corresponding author upon reasonable request.

## Abbreviations

AVF: Autologous arteriovenous fistula
AVG: Arteriovenous graft
BMI: Body mass index
CKD: Chronic kidney disease
CVC: Central venous catheter
CI: Confidence interval
DM: Diabetes mellitus
HR: Hazard ratio
HD: Hemodialysis
PAOD: Peripheral arterial occlusive disease
VA: Vascular access
