# Supplementary material for: Effects of patient age on patency of chronic hemodialysis vascular access
Source: BMC Nephrol. 2019 Nov 21;20:422. doi: 10.1186/s12882-019-1604-7 (PMC6873763; doi:10.1186/s12882-019-1604-7)
Supplement: Supplementary file 1 — Additional file 1: Table S1. Factors associated with primary VA patency in subgroup analyses of patients aged 65 years and older. This table contains the associations between clinical variables and primary VA patency in the elderly group. Table S2. Factors associated with secondary VA patency in subgroup analyses of patients aged 65 years and older. This table contains the associations between clinical variables and secondary VA patency in the elderly group. Table S3. Factors associated with primary patency in subgroup analyses of patients with AVF placement. This table contains the associations between clinical variables and primary patency in patients with AVF placement. Table S4. Factors associated with secondary patency in subgroup analyses of patients with AVF placement. This table contains the associations between clinical variables and secondary patency in patients with AVF placement. Table S5. Factors associated with primary patency in subgroup analyses of patients with AVG placement. This table contains the associations between clinical variables and primary patency in patients with AVG placement. Table S6. Factors associated with secondary patency in subgroup analyses of patients with AVG placement. This table contains the associations between clinical variables and secondary patency in patients with AVG placement. Table S7. Baseline demographics and clinical characteristics of the ≥65 study population at the time of VA placement. This table contains baseline demographic and clinical characteristics of subgroup analyses based on age (65–75 years vs. ≥75 years) in the elderly group. Table S8. Factors associated with primary VA patency in subgroup analyses of patients ≥75. This table contains the associations between clinical variables and primary patency in patients ≥75. Table S9. Factors associated with secondary VA patency in subgroup analyses of patients ≥75. This table contains the associations between clinical variables and secondary patency in patient [file 12882_2019_1604_MOESM1_ESM.docx]

**Table S1.** Factors associated with primary VA patency in subgroup analyses of patients aged 65 years and older (n = 206)

|  | Univariate | | Multivariate | |
| --- | --- | --- | --- | --- |
|  | HR (95% CI) | *P*-value | HR (95% CI) | *P*-value |
| Increased age | 1.07 (1.03–1.11) | < 0.01 | 1.02 (0.98–1.07) | 0.26 |
| Female sex | 0.99 (0.66–1.49) | 0.97 | NA | NA |
| BMI | 0.93 (0.87–0.99) | 0.02 | 0.93 (0.88–0.99) | 0.02 |
| AVF | 0.36 (0.24–0.55) | < 0.01 | 0.36 (0.24–0.54) | < 0.01 |
| Hypertension | 1.21 (0.66–2.22) | 0.539 | NA | NA |
| DM | 1.15 (0.76–1.76) | 0.504 | NA | NA |
| Smoker | 1.14 (0.71–1.84) | 0.588 | NA | NA |
| CVD | 0.98 (0.63–1.51) | 0.909 | NA | NA |
| CVA | 1.02 (0.62–1.68) | 0.926 | NA | NA |
| PAOD | 0.60 (0.26–1.37) | 0.226 | NA | NA |

AVF, autologous arteriovenous fistula; BMI, body mass index; CI, confidence interval; CVA, history of cerebrovascular accident; CVD, cardiovascular disease; DM, diabetes mellitus; HR, hazard ratio; NA, not applicable; PAOD, peripheral arterial occlusive disease; VA, vascular access

**Table S2.** Factors associated with secondary VA patency in subgroup analyses of patients aged 65 years and older (n = 206)

|  | Univariate | | Multivariate | |
| --- | --- | --- | --- | --- |
|  | HR (95% CI) | *P*-value | HR (95% CI) | *P*-value |
| Increased age | 1.13 (1.07–1.20) | < 0.01 | 1.13 (1.07–1.20) | < 0.01 |
| Female sex | 0.94 (0.50–1.92) | 0.74 | NA | NA |
| BMI | 0.91 (0.82–1.01) | 0.07 | 0.93 (0.85–1.03) | 0.17 |
| AVF | 0.36 (0.18–0.72) | < 0.01 | 0.58 (0.27–1.23) | 0.15 |
| Hypertension | 2.94 (0.70–12.28) | 0.14 | NA | NA |
| DM | 2.11 (0.98–4.52) | 0.06 | 1.82 (0.82–4.02) | 0.14 |
| Smoker | 1.63 (0.78–3.42) | 0.19 | NA | NA |
| CVD | 0.96 (0.46–2.01) | 0.91 | NA | NA |
| CVA | 1.68 (0.78–3.59) | 0.19 | NA | NA |
| PAOD | 0.28 (0.04–2.06) | 0.21 | NA | NA |

AVF, autologous arteriovenous fistula; BMI, body mass index; CI, confidence interval; CVA, history of cerebrovascular accident; CVD, cardiovascular disease; DM, diabetes mellitus; HR, hazard ratio; NA, not applicable; PAOD, peripheral arterial occlusive disease; VA, vascular access

**Table S3.** Factors associated with primary patency in subgroup analyses of patients with AVF placement (n = 521)

|  | Univariate | | Multivariate | |
| --- | --- | --- | --- | --- |
|  | HR (95% CI) | *P*-value | HR (95% CI) | *P*-value |
| Increased age | 1.01 (1.00–1.03) | 0.03 | 1.01 (1.00–1.02) | 0.08 |
| Female sex | 0.90 (0.65–1.25) | 0.53 | NA | NA |
| Forearm AVF | 0.77 (0.57–1.06) | 0.10 | 1.27 (0.93–1.74) | 0.13 |
| BMI | 1.01 (0.96–1.05) | 0.76 | NA | NA |
| Hypertension | 0.88 (0.59–1.32) | 0.53 | NA | NA |
| DM | 1.33 (0.98–1.81) | 0.07 | 1.24 (0.90–1.70) | 0.19 |
| Smoker | 0.98 (0.68–1.40) | 0.89 | NA | NA |
| CVD | 1.32 (0.88–1.99) | 0.18 | NA | NA |
| CVA | 1.12 (0.68–1.84) | 0.67 | NA | NA |
| PAOD | 0.88 (0.43–1.79) | 0.72 | NA | NA |

AVF, autologous arteriovenous fistula; BMI, body mass index; CI, confidence interval; CVA, history of cerebrovascular accident; CVD, cardiovascular disease; DM, diabetes mellitus; HR, hazard ratio; NA, not applicable; PAOD, peripheral arterial occlusive disease

**Table S4.** Factors associated with secondary patency in subgroup analyses of patients with AVF placement (n = 521)

|  | Univariate | | Multivariate | |
| --- | --- | --- | --- | --- |
|  | HR (95% CI) | *P*-value | HR (95% CI) | *P*-value |
| Increased age | 1.00 (0.98–1.01) | 0.70 | NA | NA |
| Female sex | 0.86 (0.54–1.38) | 0.53 | NA | NA |
| Forearm AVF | 0.79 (0.51–1.22) | 0.28 | NA | NA |
| BMI | 1.04 (0.99–1.10) | 0.15 | NA | NA |
| Hypertension | 0.88 (0.50–1.53) | 0.64 | NA | NA |
| DM | 1.48 (0.96–2.28) | 0.08 | 1.48 (0.96–2.28) | 0.08 |
| Smoker | 1.11 (0.68–1.83) | 0.68 | NA | NA |
| CVD | 1.65 (0.95–2.85) | 0.08 | 1.54 (0.88–2.68) | 0.13 |
| CVA | 1.11 (0.56–2.22) | 0.76 | NA | NA |
| PAOD | 0.93 (0.34–2.54) | 0.89 | NA | NA |

AVF, autologous arteriovenous fistula; BMI, body mass index; CI, confidence interval; CVA, history of cerebrovascular accident; CVD, cardiovascular disease; DM, diabetes mellitus; HR, hazard ratio; NA, not applicable; PAOD, peripheral arterial occlusive disease

**Table S5.** Factors associated with primary patency in subgroup analyses of patients with AVG placement (n = 130)

|  | Univariate | | Multivariate | |
| --- | --- | --- | --- | --- |
|  | HR (95% CI) | *P*-value | HR (95% CI) | *P*-value |
| Increased age | 1.02 (1.00–1.04) | 0.02 | 1.02 (1.00–1.04) | 0.04 |
| Female sex | 0.74 (0.47–1.16) | 0.19 | NA | NA |
| Forearm AVG | 1.04 (0.66–1.65) | 0.86 | NA | NA |
| BMI | 0.92 (0.86–0.98) | <0.01 | 0.92 (0.87–0.98) | 0.01 |
| Hypertension | 1.16 (0.58–2.34) | 0.67 | NA | NA |
| DM | 1.14 (0.72–1.82) | 0.57 | NA | NA |
| Smoker | 1.32 (0.76–2.29) | 0.33 | NA | NA |
| CVD | 0.82 (0.49–1.36) | 0.44 | NA | NA |
| CVA | 0.86 (0.46–1.59) | 0.62 | NA | NA |
| PAOD | 1.24 (0.50–3.07) | 0.65 | NA | NA |
|  |  |  |  |  |

AVG, arteriovenous graft; BMI, body mass index; CI, confidence interval; CVA, history of cerebrovascular accident; CVD, cardiovascular disease; DM, diabetes mellitus; HR, hazard ratio; NA, not applicable; PAOD, peripheral arterial occlusive disease

**Table S6.** Factors associated with secondary patency in subgroup analyses of patients with AVG placement (n = 130)

|  | Univariate | | Multivariate | |
| --- | --- | --- | --- | --- |
|  | HR (95% CI) | *P*-value | HR (95% CI) | *P*-value |
| Increased age | 1.04 (1.00–1.08) | 0.03 | 1.04 (1.00–1.07) | 0.03 |
| Female sex | 0.78 (0.38–1.62) | 0.51 | NA | NA |
| Forearm AVG | 2.27 (0.97–5.34) | 0.06 | 0.51 (0.21–1.20) | 0.12 |
| BMI | 0.91 (0.81–1.01) | 0.07 | 0.91 (0.82–1.01) | 0.08 |
| Hypertension | 1.20 (0.36–3.97) | 0.76 | NA | NA |
| DM | 1.77 (0.78–4.01) | 0.17 | NA | NA |
| Smoker | 1.40 (0.60–3.29) | 0.44 | NA | NA |
| CVD | 0.59 (0.25–1.38) | 0.23 | NA | NA |
| CVA | 1.53 (0.62–3.76) | 0.36 | NA | NA |
| PAOD | 0.50 (0.07–3.65) | 0.49 | NA | NA |
|  |  |  |  |  |

AVG, arteriovenous graft; BMI, body mass index; CI, confidence interval; CVA, history of cerebrovascular accident; CVD, cardiovascular disease; DM, diabetes mellitus; HR, hazard ratio; NA, not applicable; PAOD, peripheral arterial occlusive disease

**Table S7.** Baseline demographics and clinical characteristics of the ≥65 study population at the time of VA placement (n = 206)

|  | 65–75 years  n = 130 | ≥75 years  n = 76 | *P*-value |
| --- | --- | --- | --- |
| Age (years) | 69.1 ± 2.7 | 78.7 ± 3.3 | < 0.01 |
| Female sex | 54 (41.5) | 28 (50.0) | 0.24 |
| BMI (kg/m2) | 23.7 ± 3.4 | 22.0 ± 3.8 | < 0.01 |
| AVF | 94 (72.3) | 29 (38.2) | < 0.01 |
| Underlying diseases |  |  |  |
| Hypertension | 108 (83.1) | 73 (96.1) | 0.01 |
| DM | 79 (60.8) | 45 (59.2) | 0.83 |
| Smoker | 34 (26.2) | 10 (13.2) | 0.03 |
| CVD | 47 (36.2) | 24 (31.6) | 0.51 |
| CVA | 26 (20.0) | 17 (22.4) | 0.69 |
| PAOD | 7 (5.4) | 10 (13.2) | 0.05 |
| Medications |  |  |  |
| Anti-platelets | 76 (58.5) | 45 (60.5) | 0.77 |
| Anti-coagulants | 7 (5.4) | 4 (5.3) | 0.97 |
| Early mortality* | 8 (6.2) | 8 (10.5) | 0.26 |
| Maturation failure† | 9 (6.9) | 2 (2.6) | 0.19 |
| Early thrombosis‡ | 3 (2.3) | 7 (9.2) | 0.03 |

Continuous data are expressed as mean ± standard deviation, and categorical data as number (%).

AVF, autologous arteriovenous fistula; BMI, body mass index; CVA, history of cerebrovascular accident; CVD, cardiovascular disease; DM, diabetes mellitus; PAOD, peripheral arterial occlusive disease; VA, vascular access

* All-cause mortality within three months of VA placement but before use

† AVF maturation failure

‡ Absence of thrill and/or flow within 30 days of hemodialysis initiation via a functioning VA

**Table S8.** Factors associated with primary VA patency in subgroup analyses of patients ≥75 (n = 76)

|  | Univariate | | Multivariate | |
| --- | --- | --- | --- | --- |
|  | HR (95% CI) | *P*-value | HR (95% CI) | *P*-value |
| Increased age | 1.12 (1.02–1.23) | 0.02 | 1.09 (0.98–1.21) | 0.12 |
| Female sex | 1.06 (0.56–1.99) | 0.86 | NA | NA |
| BMI | 0.94 (0.85–1.03) | 0.19 | NA | NA |
| AVF | 0.30 (0.14–0.65) | < 0.01 | 0.31 (0.14–0.67) | < 0.01 |
| Hypertension | 0.23 (0.05–1.00) | 0.05 | 0.26 (0.06–1.21) | 0.09 |
| DM | 1.19 (0.63–2.26) | 0.59 | NA | NA |
| Smoker | 1.41 (0.50–3.97) | 0.52 | NA | NA |
| CVD | 1.03 (0.50–2.13) | 0.94 | NA | NA |
| CVA | 1.61 (0.71–3.65) | 0.26 | NA | NA |
| PAOD | 2.82 (0.87–9.19) | 0.09 | 0.38 (0.12–1.23) | 0.11 |

AVF, autologous arteriovenous fistula; BMI, body mass index; CI, confidence interval; CVA, history of cerebrovascular accident; CVD, cardiovascular disease; DM, diabetes mellitus; HR, hazard ratio; NA, not applicable; PAOD, peripheral arterial occlusive disease; VA, vascular access

**Table S9.** Factors associated with secondary VA patency in subgroup analyses of patients ≥75 (n = 76)

|  | Univariate | | Multivariate | |
| --- | --- | --- | --- | --- |
|  | HR (95% CI) | *P*-value | HR (95% CI) | *P*-value |
| Increased age | 1.20 (1.06–1.36) | < 0.01 | 1.20 (1.06–1.36) | 0.01 |
| Female sex | 0.76 (0.29–1.96) | 0.57 | NA | NA |
| BMI | 0.93 (0.81–1.07) | 0.30 | NA | NA |
| AVF | 2.63 (0.86–8.09) | 0.09 | 1.93 (0.60–6.20) | 0.27 |
| Hypertension | 1.81 (0.24–13.90) | 0.57 | NA | NA |
| DM | 0.85 (0.32–2.29) | 0.75 | NA | NA |
| Smoker | 1.89 (0.25–14.38) | 0.54 | NA | NA |
| CVD | 1.16 (0.37–3.62) | 0.80 | NA | NA |
| CVA | 0.97 (0.32–2.96) | 0.96 | NA | NA |
| PAOD | 3.55 (0.47–26.68) | 0.22 | NA | NA |

AVF, autologous arteriovenous fistula; BMI, body mass index; CI, confidence interval; CVA, history of cerebrovascular accident; CVD, cardiovascular disease; DM, diabetes mellitus; HR, hazard ratio; NA, not applicable; PAOD, peripheral arterial occlusive disease; VA, vascular access
